# The impact of a mindful compassion program for medical trainees

**DOI:** 10.1186/s12909-025-07439-6

**Published:** 2025-07-01

**Authors:** Flora Wong, Nikki Ashtiani, Raphael Cuomo, Anaheed Shirazi, Matthew Herbert, Gage Chu, Renée Lewis, Desiree Shapiro

**Affiliations:** 1 Department of Psychiatry, University of California, San Diego, CA USA; 2 School of Medicine, University of California, San Diego, CA USA; 3 Center for Excellence for Stress and Mental Health CESAMH, San Diego, CA USA; 4 VA San Diego HealthCare, San Diego, CA USA; 5 Center for Integrative Health Center for Mindfulness, University of California, San Diego, CA USA

**Keywords:** Burnout, Mindfulness, Medical Trainees, Well-being, Self-compassion, Medical Education, Connectedness

## Abstract

**Background:**

Medical training is intellectually, physically, and emotionally challenging. Trainees experience concerning rates of burnout, compassion fatigue, poor mental health outcomes, and reduced overall well-being, which may negatively impact the quality of care for patients. Mindfulness interventions help mitigate stress and reduce burnout, however, limited time, insufficient resources and training, and competing priorities are consistent barriers.

**Methods:**

A total of 22 medical trainees, comprising 17 medical students and 5 residents, participated in a 6-week mindful self-compassion training. The pilot study aimed to 1) evaluate the effect of the program on mindfulness, self-compassion, life satisfaction, connectedness, emotional intelligence, stress, and burnout among medical trainees; and 2) explore challenges and feasibility of implementing mindfulness programs in medical training. Pre- and post-intervention surveys were implemented at 6, 12, and 18 weeks.

**Results:**

Results showed significant improvements in levels of mindfulness, interconnectedness, perceived stress, life enjoyment, and compassion. However, no significant changes in rates of burnout, self-compassion, or emotional intelligence were found at any of the follow-up time points.

**Conclusions:**

Findings suggest that accessible interventions can enhance mindfulness and stress management among medical trainees. Additional research is needed to expand the sample size and scale of training.

## Introduction

Medical training is known to be rigorously challenging with extensive academic learning, long working hours, and intense emotional experiences. Levels of distress, burnout, and compassion fatigue experienced by medical trainees and physicians pose significant obstacles to the overall well-being of this population and the patients they care for and support [1, 2]. National studies have reported concerning rates of burnout and mental health conditions, which have been exacerbated by the challenges of the COVID-19 pandemic era [3, 4].

## Burnout and well-being in medical trainees

In a post-pandemic world, rates of stress and burnout have been elevated in healthcare settings. A national burnout survey revealed that 62.8% of U.S. physicians exhibited at least 1 manifestation of burnout in 2021 in comparison to 38.2% in 2020 [5]. The increased pressure experienced by medical trainees including students, interns, residents, and fellows, has been shown to decrease levels of empathy and compassion and is also related to mental health difficulties [6]. With medical trainees and physicians facing increasing demands and highly stressful situations, concern for their mental health and risk of burnout has become a central focus in recent years. Researchers have developed a growing interest in finding ways to support the mental health and well-being of trainees to prevent burnout and build resilience, all while improving the care delivered to patients and reducing costly turnover.

## Interventions to promote well-being

Various well-being and stress-reducing interventions for healthcare workers and trainees have been proposed to alleviate suffering, enhance workforce experience, and improve productivity. Although well-being is multifaceted and involves unique practices for each individual, there are evidence-based interventions available [7]. Previous studies have linked self-compassion with overall well-being and improved psychological outcomes for those who participated in self-compassion-based interventions in comparison to those who did not participate [8–10]. Compassion training has also been associated with enhanced communication skills, empathy development, and overall professional satisfaction among healthcare professionals [11, 12]. Implementation of self-compassion interventions may be a worthwhile effort to promote the well-being of healthcare trainees and professionals. When well-being is prioritized, there is potential to mitigate burnout and increase empathy and compassion in the workplace [13].

Mindfulness interventions have garnered substantial empirical support within medical training, offering a pathway to enhance well-being, alleviate stress, and foster more adept responses in clinical scenarios [14]. These interventions hold the promise of mitigating the pervasive stress endemic to healthcare environments. Current research has focused on the implementation of formal training programs such as Mindfulness Based Stress Reduction (MBSR) and Compassion Cultivation Training (CCT), with positive results among healthcare trainees [15, 16]. However, these programs are lengthy, requiring multiple hours of investment over a period of 8–10 weeks. Given the demanding nature of medical school and residency training, this can be a significant time constraint and may place further burden on trainees’ schedules while making the impact of mindful compassion training unsustainable long-term. As a result, creating programs that adhere to the limited time and resources of this population through flexible, shorter training sessions and integrating mindfulness practices into the busy schedules of healthcare trainees may allow for greater utilization and benefit [17]. Recent research has demonstrated that online delivery of self-compassion interventions is both feasible and acceptable among healthcare professionals, with participants reporting meaningful benefits in well-being comparable to in-person formats [18]. Furthermore, introducing mindfulness and compassion training earlier in medical trainees’ careers can be a pivotal opportunity to support their development of effective practices that foster well-being and emotional resilience.

The purpose of this study was to investigate the impact and feasibility of an adapted compassion training on mindfulness, self-compassion, life satisfaction, connectedness, emotional intelligence, stress, and burnout among medical trainees at UC San Diego.

## Materials and methods

Medical trainees, comprising medical students, interns, residents, and fellows, at UC San Diego, were recruited through digital flyers. Seventeen medical students and 5 residents/fellows consented to the study and were invited to participate in a 6-week training course facilitated virtually in the evenings by an expert affiliated with the UCSD Center for Mindfulness. The Mindful Self-Compassion group training was customized to accommodate busy UCSD medical trainees’ schedules and unique needs. The training is an evidence-based healthcare adaptation of the Introduction to Mindful Self-Compassion (MSC) Course, the empirically supported program of Dr. Kristin Neff at UT Austin and Dr. Chris Germer at Harvard Medical School [10]. The goal of this training was to equip participants with mindful self-compassion skills applicable in real-life scenarios with patients and colleagues. The modified MSC course spanned 75 min each week over six weeks, amounting to a total time commitment of 7.5 h. Throughout the sessions, participants engaged with the following topics: Introduction to Mindfulness; Mindfulness Practice with Discussions Regarding its Relevance in the Medical and Healthcare Setting; Understanding Self-Compassion’s Advantages and Barriers; Navigating the Inner Critic and Compassion Exercises; Exploring Multitasking and Mindfulness; Engaging in the Soft, Soothe, Allow Exercise; and Effectively Facing Intense Emotions. These sessions integrated lessons, group meditations, exercises, and consistent reflective conversations, fostering perspective exchange, community building, and mutual support.

During the first session of the Mindful Self-Compassion virtual group training, trainees were also invited to download a smartphone app developed by the investigative team, Compassion Coach, which contained reminders for engaging in compassion practices they learned during the intervention, alongside an assessment of their perceived compassion practice impact. Throughout the 6-week intervention, on every Monday, Wednesday, and Friday, participants received a morning prompt to remind them of a compassion practice, followed by an afternoon reminder, and finally, an evening assessment on the effects of their compassion practice. In total, participants received 54 prompts (3 on each Monday, Wednesday, and Friday over the 6-week period). They were also given the option to continue using the app post-study, if they desired. For additional information on Compassion Coach, see Wooldridge et al. [19].

The study featured four survey-based assessment points at 0, 6, 12, and 18 weeks. The baseline assessment included demographic details (age, sex, race, ethnicity, etc.) and standardized evaluations of compassion and emotional intelligence [20–22]. Secondary outcomes included in the assessments were burnout, quality of life, perceived stress, feeling connected to other people, and mindfulness [23–27]. Table 1 includes the validated self-report instrument used to assess the outcomes in this study. The baseline assessment, taking approximately 10–20 min, was completed remotely via an email link that directed participants to the survey. They were subsequently invited to complete identical standardized assessments immediately after the 6-week intervention (6 weeks), and at 12- and 18-weeks post-intervention. Participants were compensated $30 for each survey completed (up to $120) and up to $80 for app engagement [19].

**Table 1:** Validated survey measures utilized pre and post mindful compassion intervention

Validated Survey Measure	Description
Freiburg Mindfulness Inventory	A 14-item questionnaire designed to measure mindfulness in daily life. Each item is rated on a 4-point Likert scale (1 = rarely, 4 = almost always), with total scores ranging from 5 to 56. One item is reverse scored. Higher total scores indicate greater mindfulness [27]
Inclusion of Other in the Self (IOS) Scale	A single-item pictorial measure that assesses the perceived closeness between self and another person or group. Participants select one of seven pairs of overlapping circles, scored from 1 (no overlap) to 7 (most overlap) that best represents their relationship, with higher scores (greater overlap) indicating a stronger sense of connectedness with others [26]
Mini-Z	A 10-item questionnaire designed to assess burnout and workplace conditions in healthcare professionals. Items are rated on a 5-point Likert scale, with total scores ranging from 10 to 50. Higher scores on the burnout item indicate greater burnout. The measure also assesses job satisfaction, stress, and other aspects of work life [23]
Perceived Stress Scale (PSS)	A 10-item instrument, with each item rated on a 5-point Liker scale (0 = never, 4 = very often), with total scores ranging from 0 to 40. Higher scores reflect greater perceived stress [25]
Quality of Life Enjoyment and Satisfaction Questionnaire – Short Form (Q-LES)	A 16-item measure assessing satisfaction and enjoyment across various areas of daily functioning (i.e. household, social, etc.) and well-being. Each item is rated on a 5-point Likert scale (1 = very poor, 5 = very good), with total scores ranging from 16 to 80. Higher scores indicate greater enjoyment and satisfaction with life [24]
Santa Clara Brief Compassion (SCBC)	A 5-item measure assessing compassion toward others, particularly strangers. Each item is rated on a 7-point Likert scale (1 = not at all true of me, 7 = very true of me), with total scores ranging from 5 to 35. Higher scores reflect greater compassion for others [21]
Self-Compassion Scale –Short Form (SCS)	A 12-item questionnaire assessing how individuals respond to themselves in difficult times. Each item is rated on a 5-point Likert scale (1 = almost never, 5 = almost always), with total scores calculated as the mean of all items. Six items are reverse scored. Higher scores indicate greater self-compassion [20]
Trait Emotional Intelligence Questionnaire – Short Form (TEIQue)	A 26-item questionnaire, rated on a 7-point Likert scale (1 = completely disagree, 7 = completely agree), with total scores calculated as a mean of all items. Higher scores indicating greater emotional intelligence [22]

A summary measure for each scale was calculated for each participant at each timepoint, and then overall means were obtained for each timepoint. Paired t-tests were conducted to compare the mean scores at each follow-up timepoint (6, 12, and 18 weeks) against baseline averages.

## Results

Of the participants (*n* = 22) in this study, sixteen identified as female, 5 identified as male, 3 cisgender, 2 genderqueer, and 1 non-binary. Participants self-identified as White (32%), Asian or Asian American (36%), American Indian or Alaskan Native (5%), Black or African American (5%), some other race, ethnicity, or origin (18%), or prefer to self-describe/prefer not to say (14%). Participants were between the ages of 22–25 (23%, *n* = 5), 26–29 (36%, *n* = 8), 30–35 (32%, *n* = 7), and 36–45 (9%, *n* = 2).

Of those who participated in the study and completed the survey-based assessments, 32% completed the entire sequence of surveys at all 4 checkpoints and 27% missed one assessment checkpoint.

The average number of self-reported mindful self-compassion sessions attended was 4.25. Among the 22 participants in the study, 18 engaged in the app. Average attendance across sessions was 14 participants with a range of 11–17. Of the 18 participants who engaged in the app, there was a 51% response rate to notifications; the rate of engagement waned over time.

**Fig. 1:**
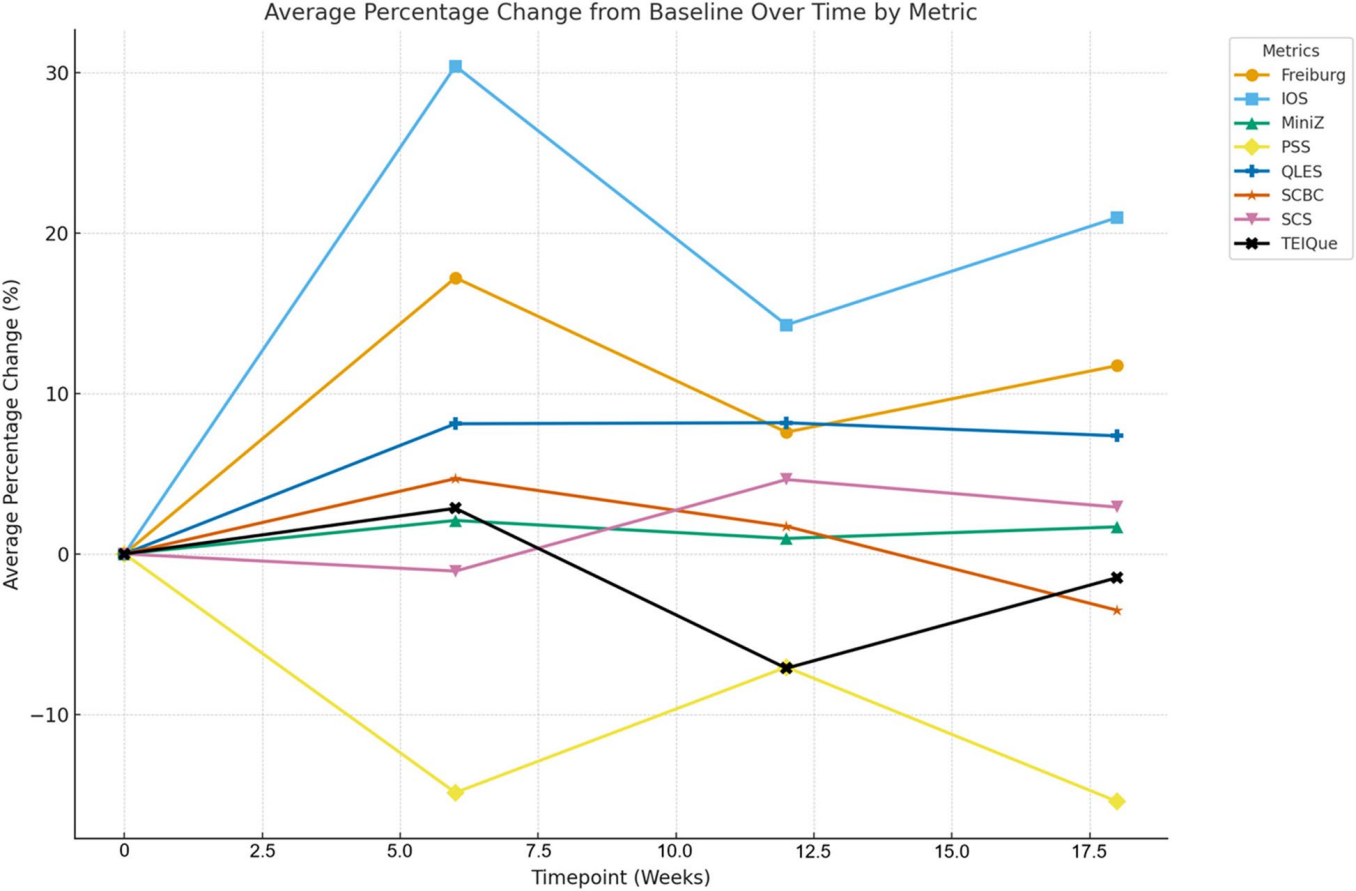
Baseline change tracking by metric

Figure 1 shows the percent change of each outcome measure over time. The Freiburg Mindfulness Inventory score showed statistically significant improvements from baseline at 6 weeks (mean = 36.28, *p* = 0.0012), which were sustained at 12 weeks (mean = 33.30, *p* = 0.0223) and 18 weeks (mean = 34.58, *p* = 0.0076). Inclusion of other in the Self Scale also demonstrated significant increases at 6 weeks (mean = 4.22, *p* = 0.0045), with 18-weeks follow-up also exhibiting significant change from baseline (mean = 3.92, *p* = 0.0189), though change at 12 weeks (mean = 3.70, *p* = 0.1157) was not statistically significant. PSS scores indicated significant reductions in perceived stress between baseline and 6 weeks (mean = 16.94, *p* = 0.0037), sustained through 12 weeks (mean = 18.50, *p* = 0.0248), and 18 weeks (mean = 16.83, *p* = 0.0018). QLES showed a significant improvement in life enjoyment measured at 18 weeks (mean = 59.25, *p* = 0.0057), though improvements were not observed for more recent follow-up measures. SCBC indicated significant improvements in compassion at 6 weeks (mean = 22.33, *p* = 0.0176), but this improvement did not appear to be sustained at more distal follow-up times. Scores for MiniZ, SCS, and TEIQue metrics did not show statistically significant changes from baseline (Table 2).

**Table 2:** Mean scores for each survey-based measure. *Denotes statistically significant differences from baseline (p < 0.05). The 6-week follow-up was immediately after the 6-week intervention

Metric	Baseline	Follow-Up		
		**6 Weeks**	**12 Weeks**	**18 Weeks**
**Freiburg**	30.95	36.28* [0.0012]	33.30* [0.0223]	34.58* [0.0076]
**IOS**	3.24	4.22* [0.0045]	3.70 [0.1157]	3.92* [0.0189]
**MiniZ**	34.67	35.39 [0.3540]	35.00 [0.8880]	35.25 [0.6945]
**PSS**	19.90	16.94* [0.0037]	18.50* [0.0248]	16.83* [0.0018]
**QLES**	55.19	59.67 [0.0805]	0.1765 [0.1765]	59.25* [0.0057]
**SCBC**	21.33	22.33* [0.0176]	21.70 [0.1039]	20.58 [1.0000]
**SCS**	3.39	3.36 [0.4140]	3.55 [0.9074]	3.49 [0.9320]
**TEIQue**	5.79	5.95 [0.4497]	5.38 [0.4063]	5.70 [0.7840]

## Discussion

This study examined the impact of delivering a virtual 6-week mindful self-compassion training program to medical students and residents at UC San Diego’s School of Medicine. This pre-post intervention study found a brief virtual training with optional app engagement reinforcing mindful compassion led to a significant and sustained improvement in mindfulness over time. Given the attendance and engagement, this intervention was determined to be feasible and well-received by trainees. The results suggest that this adapted mindfulness intervention for medical trainees had a lasting impact on participants, with improvement not only evident at the end of the program but also sustained well beyond the formal training period, which ended at the 6-week checkpoint. Similar studies have shown that mindfulness interventions can produce enduring effects on participants’ well-being [14]. This sustained effect highlights the potential of interventions promoting mindfulness and compassion to foster long-term changes in awareness and presence, critical skills for managing stress and improving well-being in high-pressure environments like medical training [28]. Learning these skills early can establish practices that positively impact healthcare workers' experiences, workplace cultures, and patient outcomes throughout their careers.

Significant increases in the participants’ sense of emotional connectedness to others were observed at 6-week and at the 18-week follow-up. However, the change at 12 weeks was not statistically significant. During the 6-week training, participants may have developed stronger interpersonal connections, both within the training group and in their personal relationships, as the group dynamic and shared experience fostered a sense of closeness. After the formal intervention ended, however, trainees may have faced challenges such as exams, rotations, match day, rigorous clinical rotations alongside lacking the sustained connection with fellow participants in the training program, which could have reduced their perceived closeness to others. As found in previous studies on mindfulness interventions, the absence of continued group support and interaction can contribute to a reduced sense of emotional connectedness [29]. This may potentially explain the lack of significant change at 12 weeks.

Furthermore, significant reductions in perceived stress were observed and sustained across all assessment checkpoint follow-ups. This intervention’s effect on reducing stress levels is particularly notable given the high-stress environment of medical training, where ongoing academic and clinical pressures often exacerbate stress levels. The persistence of these effects highlights the potential for micro-dose mindful compassion interventions to contribute to long-term stress reduction and well-being promotion in healthcare trainees.

Significant improvement in life enjoyment was observed at 18 weeks, although no significant improvement was noted at earlier follow-up points. If related to this training intervention, this result might suggest that the mindfulness intervention may have had a delayed effect on life enjoyment, with benefits becoming more apparent in the longer term. The gradual improvement at 18 weeks could reflect the time needed for the skills during the mindful compassion training and engagement with the app – such as emotional regulation, self-compassion, and greater awareness of well-being – to have a more substantial impact on participants’ overall life satisfaction. Research has shown that mindfulness and compassion interventions can lead to delayed improvements in well-being as individuals integrate these practices into their daily lives and thus long-term effects may become more evident after an initial adjustment period [15, 30].

Significant improvements in compassion towards others were observed at 6 weeks; however, these gains did not persist over longer follow-up intervals. This pattern suggests that while the intervention may have had an immediate positive effect on participants’ compassionate feelings and behaviors, the benefits may have diminished without ongoing reinforcement beyond the 6-week training program. More research is needed to determine dose and periodic booster training sessions to reinforce the mindful compassion practices initially introduced to trainees [31]. Additionally, it is important to note that the 6-week training course was an adaptation of the *Introduction to Mindful Self-Compassion (MSC)*. Additional research may elucidate how to optimize positive effects on self-compassion and compassion for others.

No statistically significant changes were found for the MiniZ Burnout Survey, Self-Compassion Scale (SCS), and Trait Emotional Intelligence Questionnaire (TEIQue). There are several possible explanations for these results that link to this study’s limitations. Burnout and emotional intelligence are complex, multifactorial constructs that may require more intensive or longer interventions to show measurable changes [32]. Emotional intelligence and self-compassion develop gradually over time with sustained practice [15, 30]. Additionally, external stressors inherent in medical training such as exams, clinical rotations, and workload pressures may have overshadowed the potential benefits of the intervention, making it harder to detect changes in these measures. Finally, variability in engagement with the mindful compassion practices, both in the sessions and through the app, could have contributed to the results. Those who did not engage regularly with the app or the mindfulness exercises outside of formal sessions may not have experienced the full benefits of the interventions. Furthermore, due to the pilot design, the current study had a small sample size. Therefore, future studies with larger samples and longer interventions may be better equipped to capture more meaningful changes in these outcomes. Notable limitations to this study include a small sample size, the absence of a control group, self-report bias, and incomplete survey responses from participants.

### Strengths and limitations

This study offers several important insights into the integration of mindful compassion practices within the context of medical training. Despite the recognized benefits of mindfulness, barriers such as time constraints, high workloads, and the rigorous demands of medical training curricula can make it difficult for trainees to engage with traditional interventions currently present in research [33]. By combining a shorter version of formal training with a user-friendly mobile app, this study offers a flexible and scalable model for mindfulness practice that can be more easily integrated into the busy lives of medical trainees. However, participation and engagement remain challenges. While participants in this study appreciated the app and reported using it to reinforce their skills, future interventions may benefit from more frequent check-ins or additional accountability measures to ensure optimal engagement. Also, as this study was conducted virtually, future research could explore in-person training options to determine optimal engagement and program format delivery.

### Future directions

Future studies should explore the integration of mindful compassion within structured healthcare settings and determine the optimal timing, duration, and structure of compassion-based interventions. Future studies could also explore the mechanisms underlying consistency of practice over time, reinforcement through digital tools, and group dynamic. Maintaining a sense of community and peer support post-intervention could further reinforce the positive effects on inclusion and emotional resilience. These interventions may be expanded across various levels of medical training to also include the preclinical years of medical school to post-graduate medical education. Furthermore, tailoring mindfulness and compassion programs to different specialties and healthcare settings could further optimize their effectiveness in addressing the unique challenges faced by different medical professionals. As empathy erosion and compassion fatigue continue to impact the medical profession, these interventions offer a promising and potentially transformative strategy for fostering mindfulness, compassion, well-being, and sustainability among future generations of physicians.

## Conclusions

In conclusion, the findings of this study suggest that brief, accessible mindful self-compassion interventions can offer significant, sustained benefits for medical trainees, enhancing mindfulness and stress management skills. The improvements observed in mindfulness, interconnectedness, perceived stress, life enjoyment, and compassion highlight the potential of integrating such programs into medical training curricula. Early introduction of such skills may foster long-term well-being and resilience in the healthcare workforce. Although no significant changes were found in burnout, self-compassion, or emotional intelligence, these measures may require both longer-term interventions to manifest noticeable change, or alternative, more targeted approaches to be effectively addressed. Future research should focus on expanding the sample size and exploring the scalability of these interventions to maximize their impact on the well-being of medical trainees, and, potentially, the quality of patient care.

## Data Availability

No datasets were generated or analysed during the current study.

## Abbreviations

MBSR: Mindfulness Based Stress Reduction
CCT: Compassion Cultivation Training
MSC: Mindful Self-Compassion
IOS: Inclusion of Other in Self
PSS: Perceived Stress Scale
QLES: Quality of Life Enjoyment and Satisfaction
SCBC: Santa Clara Brief Compassion
SCS: Self-Compassion Scale
TEIQue: Trait Emotional Intelligence Questionnaire
